# A study on the construction of a model for sustained ICS medication adherence behavior in children with asthma

**DOI:** 10.3389/fpubh.2025.1590423

**Published:** 2025-09-29

**Authors:** Li Yan, Rong Guo, Liping Wu, Chunlan Yin, Jianlan Tang

**Affiliations:** ^1^Department of Pediatric Intensive Care Unit, Children's Hospital of Chongqing Medical University, National Clinical Research Center for Child Health and Disorders, Ministry of Education Key Laboratory of Child Development and Disorders, Chongqing Engineering Research Center of Stem Cell Therapy, Chongqing, China; ^2^Department of Respiratory Medicine, Children's Hospital of Chongqing Medical University, National Clinical Research Center for Child Health and Disorders, Ministry of Education Key Laboratory of Child Development and Disorders, Chongqing Engineering Research Center of Stem Cell Therapy, Chongqing, China; ^3^Department of Nursing, Children's Hospital of Chongqing Medical University, National Clinical Research Center for Child Health and Disorders, Ministry of Education Key Laboratory of Child Development and Disorders, Chongqing Engineering Research Center of Stem Cell Therapy, Chongqing, China

**Keywords:** asthma, children, inhaled corticosteroids (ICS), sustained adherence behavior, behavioral model

## Abstract

**Objective:**

To develop a Sustained Adherence Behavior Model for inhaled corticosteroids (ICS) Medication in Children with Asthma.

**Method:**

Through literature review and semi-structured interviews, a questionnaire item pool was developed to assess the factors influencing the medication adherence behavior of asthma children using ICS. Expert consultation was conducted, involving two rounds of inquiries with experts, ultimately resulting in the development of the influence factors scale. The scale, along with the general situation survey of asthma children and the bronchial asthma medication adherence scale, was used to survey 370 asthma children who visited the outpatient department. Finally, a model of ICS medication adherence behavior for asthma children was established.

**Results:**

The positive coefficients of the two rounds of expert consultation were 90.5% and 95.2%, and the Kendall concordance coefficients were 0.384 and 0.278 (both *P* < 0.01). The Cronbach's α coefficient of the questionnaire was 0.925. The final asthma children's ICS medication adherence behavior influence factors scale consisted of 5 first-level items and 23 second-level items. Structural equation modeling analysis showed that the direct effects on medication adherence were behavioral intention (0.522), medication knowledge (0.193), and medication belief (0.181). The indirect effects were normative belief (0.161) and self-efficacy (0.140). The coefficient of determination (*R*^2^) for the influencing factors was 0.46 (*P* < 0.01).

**Conclusion:**

The behavioral model based on the Health Belief Theory effectively analyzes the factors influencing the ICS medication adherence behavior of asthma children, with the greatest impact coming from behavioral intention, followed by medication belief and medication knowledge.

## Introduction

Bronchial asthma is the most prevalent chronic respiratory disease in children, characterized by chronic airway inflammation and airway hyperresponsiveness. Childhood asthma is a globally recognized health challenge that significantly impacts children's health and is listed among the four major pediatric intractable diseases worldwide (1). It is estimated that 6 million children in our country suffer from asthma in China. Effective management of childhood asthma requires long-term, standardized, and personalized treatment with inhaled corticosteroids (ICS) (2). Medication adherence mainly refers to the degree to which a patient follows medical advice during long-term drug therapy (3). Commonly used medications include Budesonide nasal spray, Budesonide inhaler, Fluticasone propionate inhaler, and Mometasone furoate nasal spray. However, due to differences in growth and development, limited understanding of the disease, poor self-management, and inadequate inhalation techniques, children often exhibit poor medication adherence, which negatively impacts asthma control (4). According to the Global Initiative for Asthma (GINA) guidelines, 32–64% of children with asthma struggle with treatment adherence (5). This non-adherence directly affects asthma outcomes, leading to issues with physical and mental development, increased medical burden, and negative impacts on daily life. In severe cases, it raises the risk of acute exacerbations, emergency visits, and even asthma-related death (6).

Understanding and improving medication adherence requires a comprehensive theoretical framework. The Health Belief Model (HBM), a widely used conceptual model in health behavior research, provides insight into how individual beliefs about health conditions and treatment affect health-related behaviors (7). The HBM posits that individuals' likelihood of engaging in a health behavior—such as consistent ICS use—is influenced by their perceived susceptibility to the disease, perceived severity of the condition, perceived benefits of treatment, perceived barriers to adherence, cues to action, and self-efficacy. Applying the HBM to childhood asthma can help identify the factors that promote or hinder sustained adherence to ICS therapy, thus informing the development of effective interventions.

This study aims to develop a model for sustained ICS adherence in children with asthma, identify specific influencing factors, and provide insights to improve adherence. The following report presents our study.

## Methods and research content

### Establishment of the research team

The research team consisted of nine members: a chief pediatric respiratory physician, a Nursing Supervisor, a professor of statistics, two graduate nursing students, and four head nurses. The team was responsible for defining research objectives, conducting literature reviews and analyses, developing expert consultation questionnaires, selecting experts, analyzing consultation results, and refining the model for sustained ICS adherence in children with asthma. This study was approved by the hospital's ethics committee (Ethical Review Research No. 59-1 of 2020).

### Development of a questionnaire on the factors influencing sustained ICS medication adherence behavior in children with asthma

A comprehensive literature review was conducted using keyword searches across multiple databases, including the WanFang Database, VIP Chinese Science and Technology Journal Database, China Biology Medicine Journals Database, CNKI, as well as international databases such as PubMed, Embase, Web of Science, Cochrane Library, and CINAHL. Additional information was obtained from professional association websites, such as the American Association for Respiratory Care (AARC). Chinese search terms included: children, asthma, inhaled glucocorticoids, ICS, medication behavior, medication adherence, and model construction. The corresponding English search terms were: Children, Asthma, Inhaled Glucocorticoids, ICS, Medication Behavior, Medication Adherence, and Model Construction.

Based on the literature review, the research team developed and refined an interview outline through discussion. The final outline included the following questions: (1) What are the benefits of adhering to ICS inhalation? (2) What are the drawbacks of adhering to ICS inhalation? (3) Which individuals or groups influence adherence to ICS inhalation? (4) What factors promote ICS inhalation? (5) What factors hinder ICS inhalation? (6) What outcomes would you/your child hope to achieve by adhering to ICS inhalation? (7) How confident are you/your child in achieving asthma control? (8) Under what conditions would you/your child be more likely to adhere to ICS inhalation?

Semi-structured interviews were carried out by researchers trained in qualitative research methods, who maintained objectivity throughout the process. The sample size was determined by data saturation and included four asthma medical experts, four nursing experts, three management experts, four psychological experts, and ten asthmatic children with their parents from the outpatient department of Chongqing Medical University Affiliated Children's Hospital. All interviews were conducted in a quiet and comfortable environment. Participants were informed of the study's purpose and procedures, and informed consent was obtained prior to participation. Each interview lasted between 20 and 60 min, with two researchers present: one conducted the interview, while the other recorded responses in real time. Data were analyzed using Colaizzi's seven-step method and organized into themes, which were then used to generate an initial pool of questionnaire items for further study (8).

### Expert consultation

The expert consultation questionnaire consisted of three sections: The first section was an open letter explaining the purpose and methodology of the study. The second section comprised the main survey, using a 5-point Likert scale to rate the importance of each item (from 1 = Very Unimportant to 5 = Very Important), with space for additional comments and suggestions. The third section collected demographic and professional information about the experts, including their understanding, expertise, and familiarity with the consultation content.

Expert selection criteria included voluntary participation, an intermediate or higher professional title, at least 15 years of work experience, and a minimum of a bachelor's degree. A total of 20 experts were recruited from Guangdong, Shandong, Hunan, Jilin, Sichuan, Chongqing, and Yunnan provinces in China.

### Implementation of expert consultation

Between June 2021 and December 2022, the consultation questionnaire was distributed via email. After statistical analysis of the first round of responses, indicators with a mean score ≤ 3.76, a maximum score rate ≤ 74.1%, or a coefficient of variation ≥0.24 were excluded according to predetermined screening criteria. Based on expert feedback and research team discussions, a second-round questionnaire was developed and disseminated. The results of the second round demonstrated higher consensus among experts, concluding the consultation process.

### Construction of a model for sustained ICS medication adherence behavior in children with asthma

From January to March 2023, a convenience sampling method was used to recruit 370 children with asthma from the respiratory outpatient department of a tertiary children's hospital in Chongqing. The study utilized three instruments: (1) the ICS medication adherence behavior influencing factors questionnaire, (2) a general condition survey for children with asthma, and (3) a bronchial asthma medication adherence scale. For more details, see Appendices 1–3. The ICS medication adherence behavior influencing factors questionnaire was developed based on health behavior theories, including the Theory of Planned Behavior (TPB) (9, 10), Social Cognitive Theory (11, 12), and the Health Belief Model. These theories have greatly advanced our understanding of the determinants of health behaviors and the formation of behavioral intentions, thereby enhancing our ability to design effective interventions (13).

Inclusion criteria were: age ≤ 14 years; diagnosis of asthma according to the latest GINA guidelines for children; outpatient status; use of one or more of the following medications for longer than 4 weeks: beclomethasone dipropionate nasal aerosol, fluticasone propionate inhalation aerosol, budesonide inhalation aerosol, mometasone furoate nasal spray, salmeterol fluticasone powder for inhalation, or salbutamol sulfate aerosol; and provision of informed consent by both children and their families. Exclusion criteria were: comorbid severe heart, liver, kidney, or neurological diseases; a history of psychiatric disorders or current psychiatric illness; severe psychological disorders; and guardians with significant visual, auditory, speech, or cognitive impairments.

## Statistical methods

### Statistical analysis of expert consultation

For analysis of expert consultation data, double data entry and verification were performed, and data were analyzed using SPSS 25.0 software. Candidate items were retained or removed using a threshold method: the threshold for the arithmetic mean and full score rate was calculated as threshold = mean – standard deviation, retaining items above this threshold. The threshold for the coefficient of variation was calculated as threshold = mean + standard deviation, retaining items below this threshold (14). Expert engagement was assessed by the questionnaire return rate. The authority coefficient for each expert was calculated as the mean of their judgment basis and familiarity coefficients. The degree of consensus among experts was measured using Kendall's coefficient of concordance and the coefficient of variation (15).

### Statistical analysis of model construction

Measurement data with a normal distribution were described as mean ± standard deviation, while categorical data were described as frequency and rate. The relationship between patient demographic information and total asthma medication adherence scores was analyzed using independent samples t-tests and analysis of variance (ANOVA), with *post hoc* pairwise comparisons performed using the SNK-q test. Pearson correlation coefficients were used to describe correlations among factors in the theoretical model of ICS medication adherence behavior. All analyses were performed using SAS 9.4 software.

**Figure 1:**
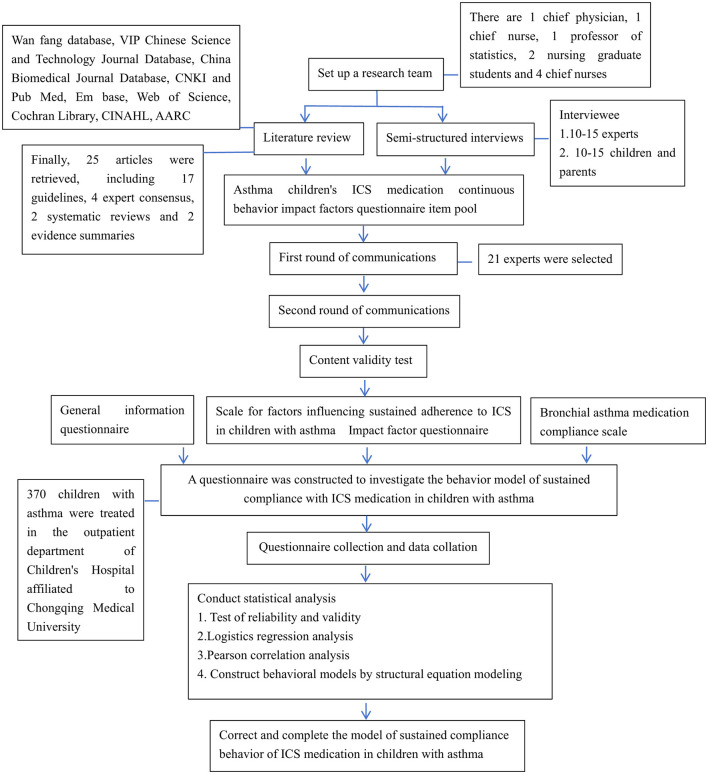
Research flow chart.

The initial structural equation model (SEM) was constructed according to the theoretical model of ICS medication adherence behavior in asthmatic children. Model parameters were estimated using the maximum likelihood method, and the bias-corrected percentile Bootstrap method (with 5000 replications) was used to calculate 95% confidence intervals for total, direct, and indirect effects. According to the criteria recommended by Daire Hooper (16), a good model fit was defined as: (1) χ^2^/degrees of freedom < 5; (2) root mean square error of approximation (RMSEA) < 0.08; (3) goodness-of-fit index (GFI) ≥ 0.90; (4) incremental fit index (IFI) ≥ 0.90; (5) confirmatory fit index (CFI) ≥ 0.90. If any path coefficient in the initial SEM had a *P-*value ≥ 0.05 or an effect opposite to theoretical expectation, the path was removed to improve model fit. All SEM analyses were conducted using Amos 24.0 software. A *P-*value less than 0.05 was considered statistically significant. The overall research process is illustrated in Figure 1.

## Results

### Literature review and semi-structured interviews

The preliminary literature search identified 4,216 articles. After screening titles and abstracts and removing duplicates, 25 articles were included for further analysis: 17 guidelines, 4 expert consensuses, 2 systematic reviews, and 2 evidence summaries. Through data synthesis and group discussion, the research team identified seven primary indicators and 39 secondary indicators, forming the initial pool of questionnaire items. These indicators encompassed normative beliefs, medication beliefs, medication environment, outcome expectations, self-efficacy, behavioral intention, and adherence behavior. Semi-structured interviews provided additional perspectives, especially from family members, including: (1) lack of awareness regarding the medication regimen; (2) fathers' concerns about potential side effects; (3) uncertainty caused by advice to discontinue medication, leading to asthma flare-ups; (4) challenges in remembering medication times; and (5) reduced confidence due to asthma attacks triggered by rhinitis. Following further team discussion, eight additional secondary indicators were incorporated. In total, the literature review and interviews yielded a preliminary questionnaire with seven primary indicators and 47 secondary indicators. For more details, see Appendix 4.

### Expert consultation

Basic information of the experts is shown in Table 1.

**Table 1:** Basic information of the experts.

**Basic information**	**Number**	**Percentage (%)**
**Education level**		
Bachelor's degree	5	23.81
Master's degree	6	28.57
Doctorate	10	47.62
**Age**		
31–40 years old	8	38.10
41–50 years old	9	42.86
Over 50 years old	4	19.04
**Professional title**		
Intermediate	5	23.81
Associate professor	9	42.86
Professor	7	33.33

Two rounds of expert consultation were conducted. In the first round, 21 questionnaires were distributed and 19 returned, resulting in a response rate of 90.5%. In the second round, 21 questionnaires were sent out, with 20 returned (response rate 95.2%). The effective response rate for both rounds was 100%. In the first consultation, 8 experts provided 29 constructive suggestions; in the second round, 6 experts contributed 15 suggestions and opinions.

The consulted experts demonstrated a high level of familiarity (coefficient 0.87), judgment basis (coefficient 0.95), and overall authority (coefficient 0.91). The coordination coefficients (Kendall's *W*) for the two consultation rounds were 0.384 and 0.278, respectively, with both reaching statistical significance (*P* < 0.001).

Following data analysis and team discussion, the first round resulted in the removal of one primary indicator and nine secondary indicators, modification of five secondary indicators, and addition of two new indicators. After the second round, four secondary indicators were deleted. The final questionnaire on factors influencing ICS adherence behavior in asthmatic children was thus established (Table 2). The Cronbach's α coefficient for this questionnaire was 0.925, indicating excellent internal consistency.

**Table 2:** Influencing factors of persistent adherence behavior to ICS medication in asthma children.

**First-level indicator**	**Second-level indicator**	**Mean**	**Coefficient of variation**	**Full score rate (%)**
**Normative beliefs**				
1.1 I believe the child should use ICS on time and in the prescribed amount		4.95 ± 0.22	0.05	100
1.2 Other caregivers believe the child should use ICS on time and in the prescribed amount		4.70 ± 0.47	0.10	100
1.3 Doctors believe the child should use ICS on time and in the prescribed amount		4.65 ± 0.58	0.13	95.0
**Medication beliefs**				
2.1 I believe using ICS on time and in the prescribed amount can control the child's asthma		4.95 ± 0.22	0.05	100
2.2 I believe using ICS on time and in the prescribed amount can help the child return to normal life		4.80 ± 0.41	0.09	100
2.3 I believe using ICS on time and in the prescribed amount can lead to drug dependence		4.75 ± 0.44	0.09	100
2.4 I believe that if the child's asthma does not attack, ICS can be discontinued		4.70 ± 0.66	0.14	90.0
2.5 I sometimes worry that long-term medication will cause adverse reactions/side effects in the child		4.80 ± 0.52	0.11	95.0
2.6 I believe not using ICS on time and in the prescribed amount is very detrimental to the child's asthma control		4.50 ± 0.83	0.18	90.0
2.7 I believe the benefits of asthma control from using ICS on time and in the prescribed amount far outweigh the side effects of the medication		4.75 ± 0.55	0.12	95.0
2.8 I am willing to spend time and effort to ensure the child uses ICS on time and in the prescribed amount		4.55 ± 0.76	0.17	95.0
**Medication environment**				
3.1 The child uses ICS on time and in the prescribed amount while at home		4.90 ± 0.31	0.06	100
3.2 The child uses ICS on time and in the prescribed amount while at school or playing outside		4.85 ± 0.37	0.08	100
3.3 We can use ICS very conveniently		4.60 ± 0.68	0.15	95.0
3.4 We can seek medical treatment very conveniently		4.40 ± 0.75	0.17	90.0
3.5 The method of using ICS by the child is simple		4.45 ± 0.89	0.20	85.0
3.6 After discharge, we can receive follow-up or guidance from medical staff		4.85 ± 0.37	0.08	100
3.7 We can obtain medication guidance through APPs, WeChat public accounts, etc.		4.50 ± 0.61	0.14	95.0
**Self-efficacy**				
4.1 I am confident in using ICS for the child on time and in the prescribed amount		4.90 ± 0.31	0.06	100
4.2 I can correctly use ICS for the child		4.85 ± 0.49	0.10	95.0
4.3 I will not let concerns about the side effects of long-term use of ICS affect the timely and proper medication for the child		4.85 ± 0.37	0.08	100
4.4 I feel exhausted about the child needing to use ICS as prescribed by a doctor for a long time		4.40 ± 0.82	0.19	90.0
**Behavioral intention**				
5.1 I will follow the advice given by doctors or other medical staff for the child		4.70 ± 0.57	0.12	95.0
5.2 I am determined to use ICS for the child on time and in the prescribed amount		5.00 ± 0.00	0.00	100
5.3 I am very likely to persist in using ICS for the child on time and in the prescribed amount in the long term		4.80 ± 0.52	0.11	95.0
**Adherence behavior**				
6.1 I remember to use ICS for the child on time and in the prescribed amount as prescribed by a doctor		4.85 ± 0.49	0.10	100
6.2 I deliberately miss doses due to concerns about side effects from corticosteroids		4.60 ± 0.75	0.16	100
6.3 The child forgets to use ICS when going to school or playing outside		4.65 ± 0.75	0.16	90.0
6.4 I stop using ICS for the child when asthma symptoms are stable		4.90 ± 0.31	0.06	100
6.5 In the past 2 weeks, I have been doing well in using ICS for the child on time and in the prescribed amount		4.65 ± 0.81	0.18	90.0

### Model construction

A total of 370 children with asthma participated in this study. Of these, 345 children (93.24%) were under 12 years of age, and 208 (56.22%) were male. The demographic characteristics and their association with medication adherence scores are shown in Table 3.

**Table 3:** The relationship between general information and total score of asthma medication adherence.

**General information**	**Number of cases**	**Asthma medication adherence total score**	***t*/*F***	***P***
**Child's age**				
< 6 years old	142 (38.38)	43.48 ± 5.61	1.135	0.323
6–11 years old	203 (54.86)	43.23 ± 6.09		
≥12 years old	25 (6.76)	41.52 ± 7.31		
**Parent's age**				
≤30 years old	70 (18.92)	44.04 ± 5.32	2.217	0.110
31–40 years old	240 (64.86)	43.3 ± 5.96		
>40 years old	60 (16.22)	41.87 ± 6.77		
**Parent's gender**				
Male	67 (18.11)	42.9 ± 6.64	−0.470	0.638
Female	303 (81.89)	43.28 ± 5.86		
**Child's gender**				
Male	208 (56.22)	43.02 ± 6.07	−0.668	0.505
Female	162 (43.78)	43.44 ± 5.93		
**Child's ethnicity**				
Han	324 (87.57)	43.24 ± 6	0.277	0.782
Ethnic minorities	46 (12.43)	42.98 ± 6.06		
**Residence**				
Urban	314 (84.86)	43.31 ± 6.06	0.789	0.431
Rural	56 (15.14)	42.63 ± 5.67		
**Marital status**				
Married	359 (97.03)	43.21 ± 6.07	0.124	0.903
Divorced + widowed	11 (2.97)	43.09 ± 3.05		
**Education level**				
Junior high school or below	72 (19.46)	42.53 ± 5.9	0.599	0.550
High school/Technical secondary school	89 (24.05)	43.49 ± 5.94		
College or above	209 (56.49)	43.32 ± 6.08		
**Monthly per capita income**				
< 5,000 yuan	154 (41.62)	42.54 ± 5.57	1.665	0.191
5,000–10,000 yuan	147 (39.73)	43.74 ± 6		
**Smoking**				
Yes	205 (55.41)	42.44 ± 6.19	−2.754	0.006
No	165 (44.59)	44.16 ± 5.64		
**Asthma family history**				
Yes	76 (20.54)	43.78 ± 6.02	0.440	0.645
No	238 (64.32)	43.09 ± 6.12		
**Time since asthma diagnosis**				
>1 year	107 (28.92)	43.75 ± 5.59	1.468	0.232
1–3 years	163 (44.05)	43.37 ± 6.04		
>3 years	100 (27.03)	42.37 ± 6.33		
**Asthma severity**				
Intermittent	149 (40.27)	42.45 ± 6.54	2.808	0.062
Mild persistent	183 (49.46)	43.95 ± 5.62		
Moderate or severe persistent	38 (10.27)	42.61 ± 5.24		
**Knowledge of medication steps**				
Not aware	131 (35.41)	43.35 ± 5.96	0.339	0.735
Aware	239 (64.59)	43.13 ± 6.04		

The results revealed that asthma medication adherence scores were significantly higher among children whose parents did not smoke compared to those whose parents did smoke (*P* < 0.05). There were no statistically significant differences in adherence scores based on child age, parent age, parent gender, child gender, child ethnicity, parent job type, place of residence, marital status, education level, monthly per capita income, family history of asthma, time since asthma diagnosis, asthma severity, or awareness of medication procedures (all *P* > 0.05).

### Factor analysis

Except for the non-significant correlation between disease nature and normative beliefs, adherence behavior was positively correlated with factors such as disease nature, triggering factors, medication knowledge, self-management, total knowledge score, normative beliefs, medication beliefs, medication environment, self-efficacy, and behavioral intention (*P* < 0.05; Table 4).

**Table 4:** Correlation analysis of asthma medication adherence with other variables in theoretical model.

**Scale**	**Adherence behavior**	**Nature of disease**	**Triggering factors**	**Medication knowledge**	**Self-management**	**Related knowledge total score**	**Normative beliefs**	**Medication beliefs**	**Medication environment**	**Self-efficacy**	**Behavioral intention**
Asthma medication adherence	1										
Nature of disease	0.222^***^	1									
Triggering factors	0.233^***^	0.480^***^	1								
Medication knowledge	0.519^***^	0.342^***^	0.367^***^	1							
Self-management	0.246^***^	0.432^***^	0.430^***^	0.520^***^	1						
Asthma medication knowledge total score	0.457^***^	0.654^***^	0.735^***^	0.844^***^	0.731^***^	1					
Normative beliefs	0.244^***^	−0.004	0.116^*^	0.226^***^	0.146^**^	0.175^***^	1				
Medication beliefs	0.495^***^	0.115^*^	0.215^***^	0.368^***^	0.106^*^	0.310^***^	0.215^***^	1			
Medication environment	0.543^***^	0.124^*^	0.211^***^	0.347^***^	0.188^***^	0.324^***^	0.158^**^	0.294^***^	1		
Self-efficacy	0.572^***^	0.124^*^	0.158^**^	0.300^***^	0.135^**^	0.245^***^	0.187^***^	0.259^***^	0.450^***^	1	
Behavioral intention	0.789^***^	0.176^***^	0.191^***^	0.426^***^	0.235^***^	0.381^***^	0.426^***^	0.433^***^	0.428^***^	0.494^***^	1

^*^P < 0.05; ^**^P < 0.01; ^***^P < 0.001.

### ICS medication adherence behavior model for children with asthma

**Figure 2:**
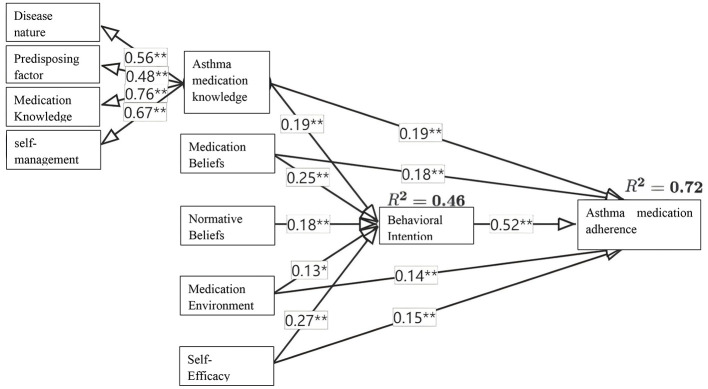
Final model of adherence behavior to ICS medication in asthma children. ^*^P < 0.01; ^**^P < 0.001.

Based on the theoretical model, an initial structural equation model (SEM) of ICS medication adherence behavior was developed. The direct path from normative beliefs to asthma medication adherence was removed due to a non-significant path coefficient (β = −0.068, *P* = 0.058). The final model is shown in Figure 2, with detailed effect values in Table 5.

**Table 5:** The total effect, direct effect, and indirect effect of asthma medication adherence.

**Indicator**	**Total effect (95% CI)**	***P***	**Direct effect (95% CI)**	***P***	**Indirect effect (95% CI)**	***P***	**Mediating effect**
Asthma medication knowledge total score	0.291 (0.186, 0.435)	<0.001	0.193 (0.110, 0.309)	<0.001	0.098 (0.058, 0.143)	<0.001	33.68%
Normative beliefs	0.161 (0.107, 0.221)	<0.001	–	–	0.161 (0.107, 0.221)	<0.001	100.00%
Medication beliefs	0.310 (0.238, 0.384)	0.001	0.181 (0.122, 0.248)	<0.001	0.128 (0.076, 0.189)	<0.001	41.29%
Medication environment	0.201 (0.109, 0.297)	<0.001	0.135 (0.067, 0.209)	<0.001	0.066 (0.014, 0.120)	0.013	32.84%
Self-efficacy	0.294 (0.151, 0.434)	<0.001	0.154 (0.039, 0.272)	0.008	0.140 (0.087, 0.197)	<0.001	47.62%
Behavioral intention	0.522 (0.458, 0.588)	<0.001	0.522 (0.458, 0.588)	<0.001	–	–	

The final model demonstrated good fit: χ^2^/d*f* = 43.274/14 = 3.091, GFI = 0.977, CFI = 0.978, IFI = 0.978, RMSEA = 0.075. All path coefficients were statistically significant (*P* < 0.05). The variables of related knowledge, normative beliefs, medication beliefs, medication environment, self-efficacy, and behavioral intention together explained 72% of the variation in adherence behavior.

The top three influencing variables were behavioral intention (total effect = 0.522), medication beliefs (0.311), and self-efficacy (0.294). Related knowledge, medication beliefs, medication environment, and self-efficacy demonstrated both direct and indirect relationships with adherence behavior via behavioral intention, with indirect effect contributions of 33.68%, 42.29%, 32.84%, and 47.62%, respectively. Normative beliefs were associated with adherence behavior only indirectly.

## Discussion

### Enrich health education methods, promote asthma medication knowledge, and build behavioral capacity

Theoretical foundations are essential for helping parents of children with asthma improve their understanding of the condition. Healthcare providers should disseminate information on asthma and inhaled medications through multiple channels to correct misconceptions and ensure that both children and caregivers recognize the dangers of asthma and learn proper medication techniques (17–19). Our findings further reinforce the importance of comprehensive education, as knowledge about medication was found to significantly influence adherence behavior in the constructed model.

Establishing strong support systems and encouraging caregivers to actively participate in disease management can enhance adherence. For example, creating WeChat groups for communication enables caregivers with good adherence to share their experiences and insights. These groups can also be used to monitor children's symptoms and asthma episodes, assess treatment progress, and promptly address inappropriate behaviors in a targeted manner (20). Regular follow-up calls to check on home health conditions—focusing on the child's sleep, diet, and emotional state—as well as providing emotional support, can help caregivers tackle challenges and maintain treatment adherence (21–23). Participation in asthma-related health education programs improves medication literacy in both patients and caregivers, leading to better adherence, enhanced asthma control, and a marked reduction in asthma attacks (24, 25).

### Improve the medication environment and enhance belief in long-term treatment

China is the world's largest tobacco consumer, with a smoking rate of 26.6% among individuals over 15 years old (26). Long-term studies have shown that smoking during pregnancy, even by grandmothers and mothers, can increase asthma risk across three generations, while paternal smoking can impact asthma incidence in their children (27). Our study shows that asthma medication adherence scores are significantly lower among children with parents who smoke, consistent with findings by Luo and Zhu (28), indicating that passive smoking has a considerable negative effect on childhood asthma. Passive smoking increases the risk of asthma in children, worsens symptoms, triggers asthma attacks, and reduces medication effectiveness. The medication environment, as identified in our model, is thus a critical factor influencing adherence, highlighting the need for a supportive home environment free from tobacco exposure.

Adherence to inhaled medication is also strongly correlated with accurate beliefs about the disease and the benefits of medication (29). In our model, medication beliefs were a key determinant of adherence (effect size = 0.311), consistent with previous findings by Li and Hao (16, 30), and supported by international research (31, 32). Positive beliefs about medication encourage integration of treatment routines into daily life, while negative beliefs may lead to non-adherence, especially if immediate benefits are not perceived (33). This underscores the importance of interventions that address misperceptions and enhance positive medication beliefs.

### Consolidate self-efficacy and strengthen the intention to continue medication behavior

Self-efficacy is a crucial protective factor that significantly influences asthma outcomes (34). High self-efficacy reflects patients' confidence in their treatment, their ability to overcome challenges, and their willingness to adhere to medical advice. Our study found a strong correlation between self-efficacy and ICS adherence (effect size = 0.294), consistent with the findings of Liu et al. (35) and other international studies (36). Behavioral intention, recognized as a strong predictor of actual behavior, was the single most influential factor in our model (effect size = 0.522), confirming its central role, as also highlighted by Han (37). In addition, our model demonstrates that medication beliefs and self-efficacy directly influence behavioral intention, which in turn mediates the effect on adherence. This aligns with research showing that psychological and behavioral factors, such as confidence and motivation, are central to sustained medication adherence (32).

To support long-term adherence, healthcare providers can enhance self-efficacy and behavioral intention through pulmonary rehabilitation exercises, regular training sessions, asthma action plans, educational brochures, and asthma diary cards. These strategies consolidate self-efficacy, reinforce behavioral intention, and help achieve sustained treatment adherence (38).

### Analysis and implications of the constructed model

The structural equation model developed in this study integrates multiple domains—knowledge, beliefs, environment, self-efficacy, and behavioral intention—based on the Health Belief Model. The model explained 72% of the variation in adherence behavior, underscoring its robustness and practical significance. Behavioral intention emerged as the most influential factor, mediating the effects of knowledge, beliefs, environment, and self-efficacy on adherence. This finding is consistent with previous research from both domestic and international settings, including studies published in Patient Preference and Adherence (31, 32, 36), which highlight the interplay between psychological, social, and environmental factors in shaping adherence behaviors among asthma patients.

### Improve the medical security platform for children with asthma to enhance medication adherence

In January 2024, the China State Council issued *The Opinions on Promoting the High-Quality Development of Children's Medical and Health Services* (39), which aims to strengthen guidance for childcare centers, improve the capabilities of schools and kindergartens in preventing and managing common childhood diseases, and enhance the management of chronic diseases in children. These policy measures provide an opportunity to implement integrated, multi-level interventions to improve asthma outcomes. A coordinated approach involving hospitals, communities, families, and schools is crucial. This includes disseminating standardized procedures for asthma medications to childcare centers, creating support networks with schools and communities, and organizing asthma self-management experience-sharing sessions. Such activities foster mutual support and learning, which, as supported by our model, can strengthen behavioral intention and self-efficacy, ultimately improving ICS adherence among children with asthma (40–43).

## Limitations

However, several limitations should be noted. First, the model was constructed and validated using data from a single center, with a sample of 370 pediatric patients from one tertiary hospital in Chongqing, which may limit both its generalizability and geographic representation. A larger and more diverse sample would enhance the persuasiveness and applicability of the findings. Second, the study adopted a cross-sectional design, precluding causal inference, and relied primarily on self-reported questionnaire data, which may introduce response bias. Third, the investigation focused only on parental perspectives regarding medication adherence, even though children's own beliefs, attitudes, and real-world behaviors—especially in school settings where direct parental supervision is not possible—may differ. Including children's perspectives in future research would make the adherence behavior influence questionnaire more comprehensive. Fourth, while family smoking was discussed, other potentially important factors such as the health status of other family members and the broader living environment were not fully explored. Fifth, the study relied on semi-structured interviews and literature review as primary data sources; incorporating objective measures (e.g., electronic monitoring of medication use or clinical outcomes) in future work would help strengthen the validity of the findings. Finally, the model did not fully capture certain external factors such as healthcare system barriers or socioeconomic status, which may also influence adherence. Future research should aim to address these limitations by validating the model in different regions, expanding sample size, collecting both parental and children's perspectives, integrating objective adherence measures, and considering additional contextual variables.

## Conclusion

In conclusion, this study developed a model of sustained ICS medication adherence behavior for children with asthma based on Health Behavior Theory, analyzing the factors influencing ICS medication adherence in these children. The study provides valuable insights into the relationships between various influencing factors and medication adherence behavior, laying a foundation for targeted health interventions. However, this phase of the study is limited to theoretical exploration. The next phase will involve integrating the behavioral model into clinical practice, applying it to the care of children with asthma and their family caregivers, and further improving medication adherence. This will provide practical evidence to support the care of children with asthma.

## Data Availability

The original contributions presented in the study are included in the article/Supplementary material, further inquiries can be directed to the corresponding author.

## References

[1] Yang YL. Application and Effectiveness Evaluation of Orems Self-Care Theory in Self-Care of School-Age Asthmatic Children. Jinzhong: Shanxi Medical University (2020).

[2] Zhang S Su L Lin R. Application of the Internet of Things management model in the management of young asthmatic children. J Nurs Sci. (2020) 35:25–8. 10.3870/j.issn.1001-4152.2020.21.025

[3] Ying Y. The Correlation Between Stigma and Medication Compliance in Adolescent Patients with Systemic Lupus Erythematosus. Beijing: Peking Union Medical College (2023).

[4] Li Y Zhao J Kan X. Clinical application study of the Chinese Children's Asthma Action Plan. Chin J Child Health Care. (2023) 31:185–8. 10.11852/zgetbjzz2022-0477

[5] Global Initiative for Asthma. Global Strategy for Asthma Management and Prevention. McLean, VA: Global Initiative for Asthma (2024).

[6] Yin J Gao Q Liu T. Asthma related death and its risk factors in children. ChinJ Appl Clin Pediatr. (2021) 36:447–52. 10.3760/cma.j.cn101070-20201128-01819

[7] Green E Murphy E Gryboski K Sweeny K Robbins M Cohen L. The Health Belief Model. (2020). p. 211–214.

[8] Ming L. Application of the seven steps of Colaizzi in phenomenological research data analysis. Nurs J. (2019) 34:90–2. 10.3870/j.issn.1001-4152.2019.11.09038228012

[9] Anderson CN Noar SM Rogers BD. The persuasive power of oral health promotion messages: a theory of planned behavior approach to dental checkups among young adults. Health Commun. (2013) 28:304–13. 10.1080/10410236.2012.684275 22742562

[10] Dutta-Bergman MJ. Theory and practice in health communication campaigns: a critical interrogation. Health Commun. (2005) 18:103–22. 10.1207/s15327027hc1802_1 16083406

[11] Bandura A. Toward a psychology of human agency: pathways and reflections. Perspect Psychol Sci. (2018) 13:130–6. 10.1177/1745691617699280 29592657

[12] Lin CY Updegraff JA Pakpour AH. The relationship between the theory of planned behavior and medication adherence in patients with epilepsy. Epilepsy Behav. (2016) 61:231–6. 10.1016/j.yebeh.2016.05.030 27390026

[13] Morris PV Flett RA Woodward LJ Anderson V Kennedy MA Milne BJ. Personality makes a difference:attachment orientation moderates theory of planned behaviorprediction of cardiac medication adherence. J Pers. (2017) 85:867–87928. 10.1111/jopy.12294 27884040

[14] Ding L Yan T Wu W Zhuang Z Li M. A study on the evaluation questionnaire of the training effect of rehabilitation medicine group standards based on Delphi method. Chin J Rehab Med. (2023) 38:805–9. 10.3969/j.issn.1001-1242.2023.06.015

[15] Chao L. Origin of Delphi Method and its Application in Biomedical Field. Jinzhong: Shanxi Medical University (2021).

[16] Li S Li P Sun H Hu W Hu S Chen Y. Medication belief is associated with improved adherence to exclusive enteral nutrition in patients with Crohn's disease. Patient Prefer Adherence. (2021) 15:2327–34. 10.2147/PPA.S330842 34703215 PMC8528542

[17] Hu H Yin Y Dong J Mao Z Hu P Song F. An individualized medication knowledge base for bronchial asthma was constructed based on the ‘Guidelines for Primary Care of Bronchial Asthma (Practice Edition 2018)'. Chin J Gener Pract. (2022) 25:1181–1185. 10.12114/j.issn.1007-9572.2022.0147

[18] Makrinioti H Fainardi V Bonnelykke K Custovic A Cicutto L Coleman C. European Respiratory Society statement on preschool wheezing disorders: updated definitions, knowledge gaps and proposed future research directions. Eur Respir J. (2024) 64:00624-2024. 10.1183/13993003.00624-2024 38843917

[19] Maciag MC Phipatanakul W. Prevention of Asthma: Targets for Intervention. Chest. (2020) 158:913–22. 10.1016/j.chest.2020.04.011 32330461 PMC7478233

[20] Gu Z Wang X Gu Q Xu W Fan J Fan J. Investigation on the standardization of medication for asthmatic children based on wechat and analysis of their medication guidance needs. J Guangdong Pharm Univ. (2022) 38:28–33. 10.16809/j.cnki.2096-3653.2022040603

[21] Wang X Wang J Qu W Lan Y Han B. Analysis of the knowledge awareness rate, medication adherence, and influencing factors among parents of children with asthma. Modern Biomed Adv. (2019) 19:3271–327. 10.13241/j.cnki.pmb.2019.17.014

[22] Shin YH Hwang J Kwon R Lee SW Kim MS Shin JI. Global, regional, and national burden of allergic disorders and their risk factors in 204 countries and territories, from 1990 to 2019: A systematic analysis for the Global Burden of Disease Study 2019. Allergy. (2023) 78:2232–54. 10.1111/all.15807 37431853 PMC10529296

[23] Heping F Juan L Luo R Enmei L. Age-related differences in IgE between childhood and adulthood allergic asthma: analysis of NHANES 2005–2006. World Allergy Organ. (2023) 16:100842. 10.1016/j.waojou.2023.100842 38213391 PMC10782400

[24] Li K. Construction and application of an intervention program for self-management of asthmatic children in school age based on the Knowledge-Attitude-Behavior theory (Master's thesis). Qingdao University, Qingdao, China (2022).

[25] Li L. The impact of outpatient health education on medication adherence and treatment outcomes in patients with bronchial asthma. Jinzhong: Shanxi Medical University (2023).

[26] Sun D Pang Y Lyu J Li L. Current progress and challenges to tobacco control in China. China CDC Wkly. (2022) 4:101–5. 10.46234/ccdcw2022.020 35186379 PMC8844520

[27] Avşar TS McLeod H Jackson L. Health outcomes of smoking during pregnancy and the postpartum period: an umbrella review. BMC Preg Childbirth. (2021) 21:254. 10.1186/s12884-021-03729-1 33771100 PMC7995767

[28] Luo X Zhu X. Guiyang area 400 cases of asthmatic childrens condition control status and influencing factors analysis. Chin J Child Health Care. (2017) 25:711–4. 10.11852/zgetbjzz2017-25-07-18

[29] Garcia-Pachon E Grau-Delgado J Baeza-Martínez C Zamora-Molina L Galán-Negrillo M Beléndez-Vázquez M. Patients' beliefs about medicines and adherence to inhalers. Open Respir Arch. (2024) 6:100322. 10.1016/j.opresp.2024.100322 38682071 PMC11053313

[30] Hao X Xie F Yan X. The therapeutic effect of budesonide on childhood asthma and its influence on growth and development 1 to 2 years after the treatment. J Pharm Service. (2021) 39:479–82. 10.12206/j.issn.1006-0111.202101013

[31] Mu X Yin C He X Li H Gong Y Wei W. Correlation between patients' medication adherence and their psychological contract with hospital pharmacists. Patient Prefer Adherence. (2020) 14:1605–13. 10.2147/PPA.S264026 32943852 PMC7478916

[32] Gu Y Mu X Zhang Y Tang Y Zhang T Tang F. The effect of patients' psychological contract with pharmacists on medication adherence: a qualitative study. Patient Prefer Adherence. (2023) 17:547–55. 10.2147/PPA.S402820 36896269 PMC9990503

[33] Hao H. Application of Greens Health Education Model in Improving Medication Adherence of Patients with Chronic Obstructive Pulmonary Disease. Yangzhou: Yangzhou University (2023).

[34] Jia Y. The Impact of Parenting Styles on Asthma Control in School-Age Children The Mediating Role of General Self-Efficacy and Medication Adherence. Shandong: Shandong University (2022).

[35] Liu Q Tao J Hu H. The impact of asthma patients perceived control on adherence to corticosteroid inhalation therapy. J Nurs Sci. (2017) 32:27–31. 10.3870/j.issn.1001-4152.2017.05.027

[36] Zhang J Yin C Li H Wei W Gong Y Tang F. Application of once-monthly self-reported ACT questionnaire in management of adherence to inhalers in outpatients with asthma. Patient Prefer Adherence. (2020) 14:1027–36. 10.2147/PPA.S176683 32606619 PMC7311206

[37] Han Y. Research on the Influencing Mechanism of Vaccine Hesitancy Integrating the Theory of Planned Behavior and Protection Motivation Theory. Nanjing: Southeast University (2022).

[38] National Clinical Research Center for Respiratory Diseases CMAPBRGACG, China Medical Education Association Pediatric Professional Committee, Chinese Medical Doctor Association Respiratory Physicians Branch Pediatric Respiratory Work Committee, Chinese Research Hospital Society Pediatrics Professional Committee, China Non-public Medical Institutions Association Pediatric Professional Committee, China Chinese Medicine Association Children's Health. Expert consensus on the clinical application of the chinese children's asthma action plan. Chin J Pract Pediatr. (2021) 36:484–90. 10.3760/cma.j.cn101070-20210310-00290

[39] National Health Commission. Opinions on Promoting the High-Quality Development of Childrens Medical and Health Services. (2024). Available online at: https://www.gov.cn/zhengce/zhengceku/202401/content.6925268.htm (Accessed January 2, 2024).

[40] Zhong T Jiang Y Ou Y. Evaluation of the use and medication compliance of inhaled glucocorticoids in children with asthma. Clin J Pulmonol. (2020) 25:697–701. 10.3969/j.issn.1009-6663.2020.05.011

[41] Yuanyuan F. The impact of targeted nursing intervention on the treatment effect and medication compliance of children with asthma. Mod Med. (2020) 48:417–21. 10.3969/j.issn.1671-7562.2020.03.02937805379

[42] Kunling S. Controlling asthma, cherishing life, and implementing the Chinese Children's Asthma Action Plan. Chin J Pract Pediatr. (2021) 36:402–4. 10.3760/cma.j.cn101070-20201217-01904

[43] Huang Z Mei Y Liu Z. Potential profile analysis of pre-hospital delayed behavioral intention in high-risk population of stroke. J Nurs Sci. (2023) 38:15–9. 10.3870/j.issn.1001-4152.2023.24.015

