# Supplementary material for: A study on the construction of a model for sustained ICS medication adherence behavior in children with asthma
Source: Front Public Health. 2025 Sep 29;13:1590423. doi: 10.3389/fpubh.2025.1590423 (PMC12517064; doi:10.3389/fpubh.2025.1590423)
Supplement: Supplementary file 1 [file Data_Sheet_1.docx]

**Appendix 1**

Questionnaire on factors affecting glucocorticoid medication compliance

in children with asthma

| Standardize beliefs | 1. I think children should be given glucocorticoids on time and in quantity | 1. Very agree 2. Agree 3. Neutral 4. Disagree 5. Very disagree |
| --- | --- | --- |
|  | 2. Other caregivers believe that the child should be given glucocorticoids on time and in adequate amounts | 1. Very agree 2. Agree 3. Neutral 4. Disagree 5. Very disagree |
|  | 3. Doctors believe that children should inhale glucocorticoids on time and in quantity | 1. Very agree 2. Agree 3. Neutral 4. Disagree 5. Very disagree |
|  | 4. Teachers believe that children should inhale glucocorticoids on time and in quantity | 1. Very agree 2. Agree 3. Neutral 4. Disagree 5. Very disagree |
|  | 5. Friends, relatives or colleagues whose children are in this situation will give their children the right amount of glucocorticoids on time | 1. Very agree 2. Agree 3. Neutral 4. Disagree 5. Very disagree |
| The belief in medication | 1.I think timely and adequate inhalation of glucocorticoids can control children's asthma | 1. Very agree 2. Agree 3. Neutral 4. Disagree 5. Very disagree |
|  | 2. I think the child can return to normal life by inhaling glucocorticoids on time and in quantity | 1. Very agree 2. Agree 3. Neutral 4. Disagree 5. Very disagree |
|  | 3. I think that inhaling glucocorticoids on time and in quantity will make children develop drug dependence | 1. Very agree 2. Agree 3. Neutral 4. Disagree 5. Very disagree |
|  | 4. I think children with asthma can not inhale glucocorticoids when they are not having an attack | 1. Very agree 2. Agree 3. Neutral 4. Disagree 5. Very disagree |
|  | 5. I sometimes worry about the adverse reactions/side effects of long-term medication on my child | 1. Very agree 2. Agree 3. Neutral 4. Disagree 5. Very disagree |
|  | 6. I don't think occasional interruptions in the process of inhaled glucocorticoids will affect the treatment effect | 1. Very agree 2. Agree 3. Neutral 4. Disagree 5. Very disagree |
|  | 7. I think the benefits of inhaled glucocorticoids on time and in quantity far outweigh the side effects of the drugs | 1. Very agree 2. Agree 3. Neutral 4. Disagree 5. Very disagree |
|  | 8.I am willing to spend time and energy urging my child to inhale glucocorticoids on time and in quantity | 1. Very agree 2. Agree 3. Neutral 4. Disagree 5. Very disagree |
| Medication environment | 1. Children will inhale glucocorticoids on time and in quantity while at home | 1. Very agree 2. Agree 3. Neutral 4. Disagree 5. Very disagree |
|  | 2. Children inhale glucocorticoids in a timely and measured manner during school or when playing outside | 1. Very agree 2. Agree 3. Neutral 4. Disagree 5. Very disagree |
|  | 3. We can easily access inhaled glucocorticoids (auxerone, dubo, salbutamol, etc.) | 1. Very agree 2. Agree 3. Neutral 4. Disagree 5. Very disagree |
|  | 4.We can get medical treatment very easily | 1. Very agree 2. Agree 3. Neutral 4. Disagree 5. Very disagree |
|  | 5. When it comes to glucocorticoids, we don't have to worry too much about money | 1. Very agree 2. Agree 3. Neutral 4. Disagree 5. Very disagree |
|  | 6. The use of inhaled glucocorticoids for children is simple and easy to master | 1. Very agree 2. Agree 3. Neutral 4. Disagree 5. Very disagree |
|  | 7. We can get guidance from medical staff whenever we have questions | 1. Very agree 2. Agree 3. Neutral 4. Disagree 5. Very disagree |
|  | 8. Asthma management APP and other software can help me keep giving glucocorticoids to my child | 1. Very agree 2. Agree 3. Neutral 4. Disagree 5. Very disagree |
| self efficacy | 1. I am confident that I will give my child glucocorticoids on time and in quantity | 1. Very agree 2. Agree 3. Neutral 4. Disagree 5. Very disagree |
|  | 2. I can teach my child to use inhaled glucocorticoids correctly | 1. Very agree 2. Agree 3. Neutral 4. Disagree 5. Very disagree |
|  | 3. I will not delay the timely and adequate inhalation of glucocorticoids because I am worried about the side effects of long-term glucocorticoids on children | 1. Very agree 2. Agree 3. Neutral 4. Disagree 5. Very disagree |
|  | 4. I feel tired that my child needs to inhale glucocorticoids for a long time according to the doctor's advice | 1. Very agree 2. Agree 3. Neutral 4. Disagree 5. Very disagree |
|  | 5. It's easy for me to give my child inhaled glucocorticoids as directed by a doctor | 1. Very agree 2. Agree 3. Neutral 4. Disagree 5. Very disagree |
| behavior disposition | 1. I can keep giving glucocorticoids to my child even if he is crying and uncooperative | 1. Very agree 2. Agree 3. Neutral 4. Disagree 5. Very disagree |
|  | 2. I was able to keep giving glucocorticoids to my child even if the short-term effects were not obvious | 1. Very agree 2. Agree 3. Neutral 4. Disagree 5. Very disagree |
|  | 3. I follow the advice of doctors or other medical personnel for my children | 1. Very agree 2. Agree 3. Neutral 4. Disagree 5. Very disagree |
|  | 4. I made up my mind to give my child glucocorticoids on time and in quantity | 1. Very agree 2. Agree 3. Neutral 4. Disagree 5. Very disagree |
|  | 5. I can adhere to the regular inhalation of glucocorticoids for a long time | 1. Very agree 2. Agree 3. Neutral 4. Disagree 5. Very disagree |
| Compliance behavior | 1. I remember giving my child glucocorticoids as prescribed by his doctor | 1. Very agree 2. Agree 3. Neutral 4. Disagree 5. Very disagree |
|  | 2. I would deliberately skip glucocorticoids because of side effects | 1. Very agree 2. Agree 3. Neutral 4. Disagree 5. Very disagree |
|  | 3. I forget to give my child glucocorticoids when they go to school or play outside | 1. Very agree 2. Agree 3. Neutral 4. Disagree 5. Very disagree |
|  | 4. I will stop giving the child glucocorticoids when asthma symptoms are stable | 1. Very agree 2. Agree 3. Neutral 4. Disagree 5. Very disagree |
|  | 5. For the past two weeks, I've been giving my child glucocorticoids on time and in quantity | 1. Very agree 2. Agree 3. Neutral 4. Disagree 5. Very disagree |
|  | 6. Children can inhale glucocorticoids on their own or with parental assistance | 1. Very agree 2. Agree 3. Neutral 4. Disagree 5. Very disagree |

**Appendix 2**

# **General information questionnaire**

| Age of child: | Year： | Sex of child: | 1. man 2. woman | Ethnicity of the child: | 1. the Han nationality 2. Minority nationalities |
| --- | --- | --- | --- | --- | --- |
| Parent age: | year ： | Parent gender: | 1. man 2. woman | Type of parental work: | 1. Fixed job (long-term and stable job)  2. Temporary work  3. Freelancing  4. Unemployed  5. Housewife/husband  6. Retirement |
| Your place of residence: | 1. Cities  2. Countryside | Your marital status: | 1. Married  2. Divorce  3. Widowhood | Your educational background: | 1. Primary school and below  2. Junior high school  3. High school/vocational high school  4. College/Bachelor's degree  5. Master degree or above |
| In the past year, your family's monthly  per capita income (including physical income and rental income) is: ______ yuan. | 1.<5000 yuan  2,500-10,000 yuan  3,100-20,000 yuan  4.> 20,000 yuan | Does anyone in your home (including yourself) smoke? | 1. Yes  2. No | Do you have a family history of asthma: | 1. Yes  2. No  3. Not clear |
| When your child was diagnosed with asthma (how long ago): | 1 <6 months  2.6 months to 1 year  3.1-3 years  4> 3 years | How severe your child's asthma is: | 1. Intermittent state  2. Mild persistence  3. Moderate duration  4. Severe persistence | Medication used in child (ICS): | 1. Sametrol tericron powder inhaler  2. Budesonide formoterol powder inhaler  3. Auxetin  4. Salbutamol sulfate aerosol  5. Changdi  other |
| Your contact information: telephone |  |  |  |  |  |

**Appendix 3**

Bronchial asthma medication compliance scale

| The nature of disease | 1. Asthma is a chronic inflammatory disease that requires long-term treatment | 1. Yes 2. No |
| --- | --- | --- |
|  | 2. Asthma is a neurological or psychological disease | 1. Yes 2. No |
|  | 3. During asthma, the muscles of the airway contract and the airway narrows | 1. Yes 2. No |
| inducing factor | 1. Asthma often strikes without warning | 1. Yes 2. No |
|  | 2. Asthma attacks are linked to emotions | 1. Yes 2. No |
|  | 3. Diet and environment can also lead to asthma attacks or exacerbations | 1. Yes 2. No |
|  | 4. Smoking or passive smoking can trigger or worsen asthma attacks | 1. Yes 2. No |
|  | 5. The number of asthma attacks will be reduced if triggers can be identified and avoided | 1. Yes 2. No |
|  | 6. When you are about to be exposed to an asthma trigger, you should wait until symptoms appear before taking the drug | 1. Yes 2. No |
|  | 7. If exercise occasionally leads to an asthma attack, you should not exercise | 1. Yes 2. No |
| Knowledge of medication | 1. Taking certain medications 10 minutes before exercise can prevent asthma attacks during exercise | 1. Yes 2. No |
|  | Although asthma cannot be cured, it can be controlled with the right medication | 1. Yes 2. No |
|  | 3. Long-term inhalation of glucocorticoids (such as salbutamol, dobo) is the most effective way to prevent asthma attacks | 1. Yes 2. No |
|  | 4. Antibiotics (such as cephalosporin, penicillin, etc.) should be used to treat asthma attacks | 1. Yes 2. No |
|  | 5. Use glucocorticoids (such as salbutamol, dobo) even when asthma is not active | 1. Yes 2. No |
|  | 6. Asthma must be treated with a bronchodilator (salbutamol) every day | 1. Yes 2. No |
|  | 7. If you have a cold or flu, you should increase your asthma medication | 1. Yes 2. No |
|  | 8. Oral medications work as quickly as inhaled medications | 1. Yes 2. No |
|  | 9. Inhalation drugs have fewer side effects than oral drugs | 1. Yes 2. No |
|  | 10. If you use a single asthma reliever (such as salbutamol) for a long time, it may not work well | 1. Yes 2. No |
| self-management | 1. If asthma does not occur, regular outpatient visits are not required | 1. Yes 2. No |
|  | 2. Regular lung function tests can monitor changes in the condition | 1. Yes 2. No |
|  | 3. The use of peak flow rate instrument monitoring can understand the change of disease earlier | 1. Yes 2. No |
|  | 4. Keep an asthma diary every day to keep track of your condition | 1. Yes 2. No |
| compliance | 1. I only take glucocorticoids when my child needs them | 1. Always 2. Often 3. Sometimes 4. Rarely 5. Never |
|  | 2. I only take glucocorticoids when the child is having trouble breathing | 1. Always 2. Often 3. Sometimes 4. Rarely 5. Never |
|  | 3. I decided to give my child a dose less of glucocorticoids | 1. Always 2. Often 3. Sometimes 4. Rarely 5. Never |
|  | 4. I try to avoid giving my child glucocorticoids | 1. Always 2. Often 3. Sometimes 4. Rarely 5. Never |
|  | 5. I would forget to give my child glucocorticoids | 1. Always 2. Often 3. Sometimes 4. Rarely 5. Never |
|  | 6. I changed the dosage for my child myself | 1. Always 2. Often 3. Sometimes 4. Rarely 5. Never |
|  | I will stop giving my child medicine for a while | 1. Always 2. Often 3. Sometimes 4. Rarely 5. Never |
|  | 8. I would only consider giving the child glucocorticoids if other treatments had failed | 1. Always 2. Often 3. Sometimes 4. Rarely 5. Never |
|  | 9. I give my child glucocorticoids before doing anything that might make the child's breathing difficult | 1. Always 2. Often 3. Sometimes 4. Rarely 5. Never |
|  | 10. I gave my child less glucocorticoids than the doctor required | 1. Always 2. Often 3. Sometimes 4. Rarely 5. Never |

**Appendix 4**

**Construction of a questionnaire on factors affecting the continuous compliance behavior of asthma children taking ICS First round of expert mail-in questionnaire**

Dear Experts,

Hello! We are the research group of factors affecting the continuous compliance behavior of ICS medication in children with asthma in the Department of Respiratory Medicine, Children's Hospital affiliated to Chongqing Medical University.

Bronchial asthma is the most common chronic airway inflammatory disease in children. According to 2015 statistics, the global asthma prevalence was 16.4%, and it is projected that by 2025, the number of children with asthma worldwide will reach 100 million. However, poor adherence to ICS (Inhaled corticosteroids) in children with asthma is a widespread issue, with various explanations for this phenomenon. Our research aims to develop a questionnaire based on health behavior theory to assess the factors influencing the sustained adherence to ICS in children with asthma, providing a scientific tool for evaluating and analyzing these factors. Additionally, we will construct a predictive model of the sustained adherence to ICS in children with asthma using structural equation modeling, offering a theoretical basis for exploring the pathways and mechanisms through which health behaviors influence medication adherence.

Health behavior theories encompass the Theory of Planned Behavior (TPB), Social Cognitive Theory, and Health Belief Model. The Theory of Planned Behavior, proposed by Ajzen, is one of the most renowned theories in social psychology that explains the relationship between attitudes and behaviors. TPB posits that behavioral intention is the most direct factor influencing behavior, which in turn is influenced by attitude, subjective norms, and perceived behavioral control. This theory has strong explanatory and predictive power and can be used for intervention. Social Cognitive Theory (SCT) suggests that there are causal relationships among individual environment, cognition, and behavior, covering nine dimensions such as self-efficacy, outcome expectations, and environmental factors. It is primarily used to intervene in physical and mental health behaviors. These theories have significantly enhanced our understanding of the factors influencing health issues and the development of behavioral intentions, thereby improving our ability to intervene in these issues. However, research on the relationship between health behavior theories and the adherence to ICS medication in children with asthma remains limited. Therefore, it is both feasible and urgent to use health behavior theories to explore the factors and mechanisms affecting the adherence to ICS medication in children with asthma, and to develop predictive models.

To better understand the factors influencing the sustained adherence to ICS medication in children with asthma, our research team, based at the Department of Respiratory Medicine, Children's Hospital Affiliated to Chongqing Medical University, has developed a questionnaire item pool by referencing domestic and international literature. This questionnaire is structured around seven dimensions: normative beliefs, medication beliefs, environment, outcome expectations, self-efficacy, behavioral intention, and adherence behavior. We have compiled the' Current Status Survey Questionnaire on Factors Influencing the Sustained Adherence to ICS Medication in Asthmatic Children.' We sincerely invite you to provide feedback on the content of this questionnaire, as your input will be crucial for our research. The study employs an expert consultation method to gather expert opinions. Each round of consultation results is summarized and analyzed before being fed back to the experts for their reference and to solicit new suggestions. We appreciate your support and guidance during your busy schedule. The questionnaire consists of two parts: the first part involves scoring the importance of the questionnaire content, and the second part includes the basic information form for experts. Please complete the relevant sections. This questionnaire is conducted anonymously, and your data will only be used for statistical analysis, not for any other purposes, and will be kept strictly confidential. Given the strict time limit for the research project, please return the completed questionnaire to our email within one week. If you have any questions, please feel free to contact us at any time

Thank you very much for your participation, look forward to your reply!


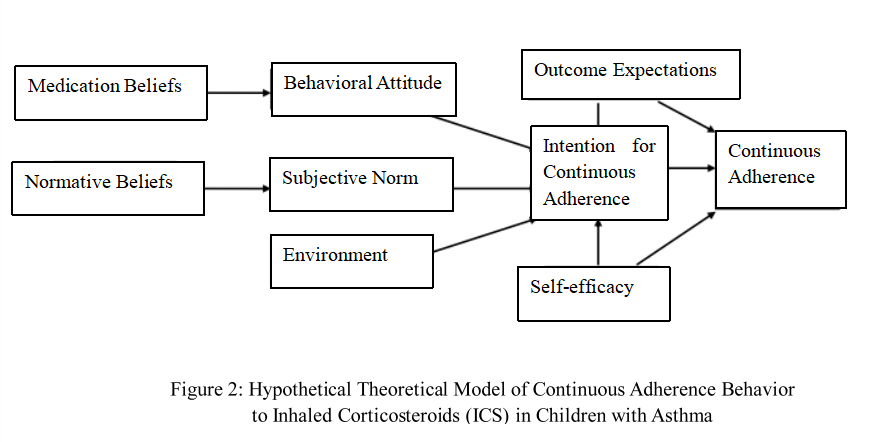


Department of Respiratory Medicine, Children's Hospital affiliated to Chongqing Medical University

Asthma children ICS medication adherence behavior influencing factors research group

January 2, 2022

contact way ：

Guo Rong, Deputy Director of the Respiratory Department of the Children's Hospital affiliated to Chongqing Medical University: 15215029996

**Part I: Questionnaire on influencing factors of continuous compliance behavior of ICS medication in asthmatic children**

**Guidance on completing the form**

**Please evaluate and score the importance of each indicator, using five levels: very important (5 points), important (4 points), general (3 points), not very important (2 points), and completely unimportant (1 point). If you have any suggestions for the indicators, please provide them in the comments section. Please do not omit or leave any items blank. We greatly appreciate your cooperation. Please mark the appropriate option with an "√"**

**Table 1 Primary candidate indicators (categorized dimensions) of factors influencing the sustained compliance behavior of ICS medication in children with asthma**

| **Classification dimensions** | **Score of the importance of the content of the item (tick in the corresponding column)** | | | | | **suggestions on revision** |
| --- | --- | --- | --- | --- | --- | --- |
|  | **very important**  **Five minutes** | **important**  **Four minutes** | **same as**  **Three minutes** | **Not too important**  **Two minutes** | **It doesn't matter at all**  **One minute** |  |
| Standardize beliefs |  |  |  |  |  |  |
| The belief in medication |  |  |  |  |  |  |
| Medication environment |  |  |  |  |  |  |
| Results expected |  |  |  |  |  |  |
| self efficacy |  |  |  |  |  |  |
| behavior disposition |  |  |  |  |  |  |
| Compliance behavior |  |  |  |  |  |  |
| **Add items** |  |  |  |  |  |  |
| **Add items** |  |  |  |  |  |  |

If you have any other comments or suggestions on Table 1, please fill in here:

**Table 2 Secondary indicators (item content) of factors influencing the sustained compliance behavior of ICS medication in children with asthma**

| **Classification dimensions** | **Item content** | **Importance of item content score (tick in the corresponding column)** | | | | | **suggestions on revision** |
| --- | --- | --- | --- | --- | --- | --- | --- |
|  |  | **very important**  **Five minutes** | **important**  **Four minutes** | **same as**  **Three minutes** | **not too**  **important**  **Two minutes** | **whole**  **unimportance**  **One minute** |  |
| **1. Standardize beliefs** | 1.1 Family and friends believe that the child is taking glucocorticoids on time and in adequate amounts |  |  |  |  |  |  |
|  | 1.2 The doctor believes that the child takes glucocorticoids on time and in adequate amounts |  |  |  |  |  |  |
|  | 1.3 If the children of friends, relatives or colleagues are in such a situation, they will give their children glucocorticoids on time and in quantity |  |  |  |  |  |  |
|  | 1.4 My spouse believes that the child should be given glucocorticoids on time and in adequate amounts |  |  |  |  |  |  |
|  | 1.5 The child's (external) grandparents believe that the child should inhale glucocorticoids on time and in quantity |  |  |  |  |  |  |
|  | 1.6 Children will inhale glucocorticoids on time and in quantity |  |  |  |  |  |  |
|  | **Add items** |  |  |  |  |  |  |
|  | **Add items** |  |  |  |  |  |  |
| **2. Beliefs about medication** | 2.1 I believe that timely and adequate inhalation of glucocorticoids can control children's asthma |  |  |  |  |  |  |
|  | 2.2 I don't think it will make the child's condition worse if he inhales glucocorticoids on time and in quantity |  |  |  |  |  |  |
|  | 2.3 I think that the timely and quantitative inhalation of glucocorticoids can restore the normal life of children |  |  |  |  |  |  |
|  | 2.4 I think children who inhale glucocorticoids on time and in quantity will develop drug dependence |  |  |  |  |  |  |
|  | 2.5 Corticosteroids don't work as well as they think they do |  |  |  |  |  |  |
|  | 2.6 Most drugs have side effects |  |  |  |  |  |  |
|  | 2.7 I think children can avoid glucocorticoids if they are asymptomatic |  |  |  |  |  |  |
|  | 2.8 I think the child will still have asthma symptoms if he or she inhales glucocorticoids on time and in quantity |  |  |  |  |  |  |
|  | 2.9 The use of glucocorticoids by my child has brought inconvenience to my life |  |  |  |  |  |  |
|  | 2.10 I sometimes worry that long-term use of drugs will cause adverse reactions in children |  |  |  |  |  |  |
|  | **Add items** |  |  |  |  |  |  |
|  | **Add items** |  |  |  |  |  |  |
| **3. Medication environment** | 3.1 During hospitalization, the child will inhale glucocorticoids on time and in quantity |  |  |  |  |  |  |
|  | 3.2 Children will inhale glucocorticoids on time and in quantity during home stay |  |  |  |  |  |  |
|  | 3.3 During school, children will inhale glucocorticoids on time and in quantity |  |  |  |  |  |  |
|  | 3.4 Under my supervision, the child will inhale glucocorticoids on time and in quantity |  |  |  |  |  |  |
|  | 3.5 Children who are alone will take in glucocorticoids on time and in quantity |  |  |  |  |  |  |
|  | 3.6 We have easy access to glucocorticoids |  |  |  |  |  |  |
|  | 3.7 We can get help in time when we need medication guidance |  |  |  |  |  |  |
|  | 3.8 In the process of using glucocorticoids, we don't have to worry too much about money |  |  |  |  |  |  |
|  | **Add items** |  |  |  |  |  |  |
|  | **Add items** |  |  |  |  |  |  |
| **4. Expected results** | 4.1 If glucocorticoids are inhaled on time and in quantity, children will visit the doctor less often |  |  |  |  |  |  |
|  | 4.2 If glucocorticoids are inhaled on time and in quantity, children will have fewer asthma symptoms |  |  |  |  |  |  |
|  | 4.3 If the glucocorticoids are inhaled on time and in quantity, the child will go to school normally |  |  |  |  |  |  |
|  | 4.4 If glucocorticoids are inhaled on time and in quantity, the child will return to normal life |  |  |  |  |  |  |
|  | 4.5 If glucocorticoids are inhaled on time and in quantity, the child's asthma will be controlled |  |  |  |  |  |  |
|  | 4.6 If glucocorticoids are inhaled on time and in quantity, the child's condition will not improve significantly |  |  |  |  |  |  |
|  | **Add items** |  |  |  |  |  |  |
|  | **Add items** |  |  |  |  |  |  |
| **5. Self-efficacy** | 5.1 I am confident that glucocorticoids will be administered to the child on time and in adequate quantities |  |  |  |  |  |  |
|  | 5.2 I know that timely and adequate inhaled glucocorticoids are effective in controlling my child's asthma symptoms |  |  |  |  |  |  |
|  | 5.3 I can teach my child to use inhalation drugs correctly |  |  |  |  |  |  |
|  | 5.4 I can inhale glucocorticoids for my children on time and in quantity without being reminded by others |  |  |  |  |  |  |
|  | 5.5 I will not worry about the side effects of long-term glucocorticoid inhalation on children, and will inhale glucocorticoid in time and in quantity |  |  |  |  |  |  |
|  | 5.6 The child took glucocorticoids as prescribed by the doctor, and I felt tired |  |  |  |  |  |  |
|  | 5.7 How easy it is to give glucocorticoids to children as prescribed by a doctor |  |  |  |  |  |  |
|  | **Add items** |  |  |  |  |  |  |
|  | **Add items** |  |  |  |  |  |  |
| **6. Behavioral intention** | 6.1 I plan to give my child glucocorticoids on time and in accordance with the doctor's advice |  |  |  |  |  |  |
|  | 6.2 How likely do you think it is that you will give your child glucocorticoids on time and in adequate amounts |  |  |  |  |  |  |
|  | 6.3 Looking back, would you have followed the advice of a doctor or other medical personnel given to your child? |  |  |  |  |  |  |
|  | 6.4 I made up my mind to give glucocorticoids to my child on time and in quantity |  |  |  |  |  |  |
|  | 6.5 In the past two weeks, how often and how much glucocorticoids you have given your child |  |  |  |  |  |  |
|  | **Add items** |  |  |  |  |  |  |
|  | **Add items** |  |  |  |  |  |  |
| **7. Compliance behavior** | 7.1 I will remember to give my child glucocorticoids as prescribed by the doctor on time and in quantity |  |  |  |  |  |  |
|  | 7.2 Difficulty remembering the time and dose of inhaled glucocorticoids |  |  |  |  |  |  |
|  | 7.3 I will intentionally omit the administration of glucocorticoids due to adverse reactions caused by glucocorticoids |  |  |  |  |  |  |
|  | 7.4 Children forget to inhale glucocorticoids when they go to school or play outside |  |  |  |  |  |  |
|  | 7.5 I will stop inhaled glucocorticoids in children when asthma symptoms are stable |  |  |  |  |  |  |
|  | **Add items** |  |  |  |  |  |  |
|  | **Add items** |  |  |  |  |  |  |

If you have any other comments or suggestions on Table 2, please fill in here:

**The second part inquires about the basic information of experts**

I. General information about experts

Please fill in the following information or tick "√" in the corresponding box

1. Sex: ▢ Male ▢ Female
2. age ：
3. Highest degree: ▢ junior college ▢ university ▢ master's degree ▢ doctor's degree ▢ other
4. Professional title: ▢ intermediate professional title ▢ associate senior professional title ▢ senior professional title ▢ other
5. research area ：
6. Engaged in pediatric asthma clinical work for years

2. Investigation of the authority of experts

To understand the expert's familiarity with the content of the form and the expert's judgment ability, please click "√" in the appropriate option column

The expert's familiarity with the content of the form

| know…well | More familiar | Generally familiar | be unfamiliar with | Not very familiar with it |
| --- | --- | --- | --- | --- |
|  |  |  |  |  |

Expert judgment ability in filling out forms

| Judgment criteria | The extent to which expert judgment is influenced | | |
| --- | --- | --- | --- |
|  | big | centre | small |
| clinical experience |  |  |  |
| theoretical analysis |  |  |  |
| Understanding of domestic and foreign situations |  |  |  |
| intuitive feeling |  |  |  |
